# Impact of Traces of Hydrogen Sulfide on the Efficiency of Ziegler–Natta Catalyst on the Final Properties of Polypropylene

**DOI:** 10.3390/polym14183910

**Published:** 2022-09-19

**Authors:** Joaquín Hernández-Fernández, Heidi Cano, Miguel Aldas

**Affiliations:** 1 Chemistry Program, Department of Natural and Exact Sciences, San Pablo Campus, University of Cartagena, Cartagena 130015, Bolivar, Colombia; 2 Department of Civil and Environment Engineering, Universidad de la Costa, Calle 58 #55-66, Barranquilla 080002, Atlántico, Colombia; 3 Departamento de Ciencia de Alimentos y Biotecnología, Facultad de Ingeniería Química y Agroindustria, Escuela Politécnica Nacional, Quito 170517, Ecuador

**Keywords:** hydrogen sulfide, ligands, polypropylene, catalyst, degradation

## Abstract

Sulfur compounds are removed from propylene through purification processes. However, these processes are not 100% effective, so low concentrations of compounds such as H_2_S may be present in polymer-grade propylene. This article studies the effects of H_2_S content on polypropylene polymerization through the controlled dosage of this compound with concentrations between 0.07 and 5 ppm and its monitoring during the process to determine possible reaction mechanisms and evaluate variations in properties of the material by TGA, FTIR, MFI, and XDR analysis. It was found that the fluidity index increases directly proportional to the concentration of H_2_S. In addition, the thermo-oxidative degradation is explained by means of the proposed reaction mechanisms of the active center of the Ziegler–Natta catalyst with the H_2_S molecule and the formation of substances with functional groups such as alcohol, ketones, aldehydes, CO, and CO_2_ by the oxidation of radical complexes. This study shows for the first time a reaction mechanism between the active center formed for polymerization and H_2_S, in addition to showing how trace impurities in the raw materials can affect the process, highlighting the importance of optimizing the processes of removal and purification of polymer-grade materials.

## 1. Introduction

Raw materials are of great importance during the synthesis of polypropylene on an industrial scale. In this process, catalysts (Ziegler–Natta (ZN) catalyst), co-catalysts (Triethyl aluminum (TEAL)), monomers (propylene), selectivity agents, and gases (hydrogen and nitrogen) are used [1,2] in order to ensure that one of them has a transition metal in its structure or can act as a Lewis acid [1,2]. The production of polypropylene consists of several stages, beginning with the initiation reaction where the activation occurs, which would be the active center and the double bond of propylene [3]. Next follows the polymerization stage where the chain of monomers is formed and finally the termination step, where the chain formation reaction is terminated. Because the catalyst (titanium (IV) chloride) is a strong Lewis acid, it tends to form several complexes with a wide variety of ligands [4,5]. For this reason, the effect of impurities that can react with it has been studied and quantified [6,7,8,9,10,11,12].

The chemical compound of inorganic nature, hydrogen sulfide (H_2_S), is composed of one sulfur atom and two hydrogen atoms in a bent geometry analogous to the water molecule, in which the two hydrogen atoms join the sulfur at an angle of 100°, and is a weak acid [13,14,15]. The H_2_S compound has the ability to bond with transition metals (M), forming metal sulfides that are generally insoluble. The transition metals that have an affinity for the formation of these complexes are those that have partially filled d orbitals and empty s and p orbitals [16,17]. Unlike the above, sulfur has available d orbitals that allow it to form d-d bonds, which can also occur in early transition metals in low oxidation states [15], on which polarizabilities and the number of lone pairs must be considered, on which the effectiveness of accepting electrons will depend [18,19]. The formation of these compounds in the catalysis of polymeric materials is of great interest due to their organometallic characteristics, such as their ability to form vacant coordinated sites to understand the reactivity and behavior in catalysis [20,21]. Generally, for these studies, analytical techniques such as spectrophotometry and X-rays are used to understand the interaction of sulfuric ligands with transition metals and determine the oxidation of the compounds [13,22].

H_2_S can be present in propylene due to failures during its production. Propylene is produced through the catalytic cracking of hydrocarbons, which contain organic sulfur compounds that are removed via desulfurization processes such as hydrotreatment, absorption, or the use of sulfide-reducing catalysts, to name a few [23,24,25,26]. These treatments are not completely effective, so traces of unremoved compounds become impurities in the oil-derived compounds. Usually, the presence of these sulfuric compounds in LPG is studied to improve removal processes [27]. However, their effects on the polymerization of LPG derivatives are scarce and do not allow us to understand their interactions with the compounds used during polymerization, such as catalysts and co-catalysts, which would allow us to understand how the final material obtained can be affected.

Polymerization determines the properties of the polymer obtained. If it is affected by the presence of unwanted compounds; these effects can be seen in the final properties of the material [28,29]. In the case of sulfides, it is important to consider the polymerization process to determine how it influences the series of reactions that occur during polymerization [30]. In some cases, it is considered a polymerization inhibitor, and in others, an opportunity to control polymerization [31,32,33]. In this research, the effects that H_2_S has on the efficiency of the Ziegler–Natta catalyst and on the thermal and physicochemical properties of PP obtained are evaluated, and its purpose is to propose reaction mechanisms that allow an understanding of the interaction of H_2_S with the raw materials used for PP production. For this purpose, four samples of PP with different doses of H_2_S are synthesized; the components formed in each stage of production and the final properties of the resin obtained will be evaluated.

## 2. Materials and Methods

### 2.1. Reagents

H_2_S with a purity of 99.99% from Merk, Germany, was used, and it was mixed with the balance of liquefied petroleum gas (LPG) in concentrations ranging from 0.07 to 5 ppm via an in-line diffuser. For the production of PP, polymer-grade propylene (Shazand Petrochemical, Arak, Iran) was used, along with a fourth-generation Ziegler–Natta catalyst with MgCl_2_ support, and as an internal donor, diisobutyl phthalate (DIBP) supplied by Sudchemie, Germany; triethylaluminum (TEAL) is 98% pure from Merk, Germany, diluted in n-heptane. Cyclohexyl methyl dimethoxysilane (CMDS) from Merk, Germany, as an external donor, and hydrogen and nitrogen gases were also used [34,35].

### 2.2. Polymerization

**Figure 1:**
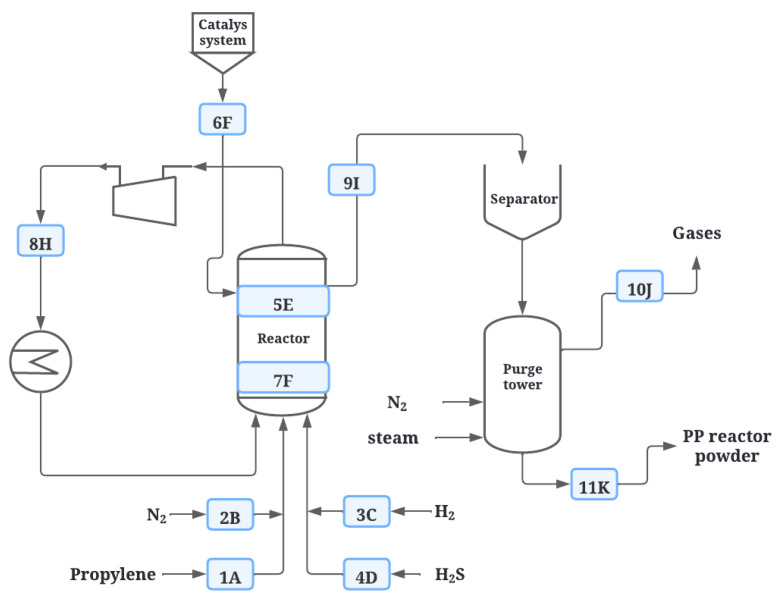
PP production scheme.

The gas-phase polymerization process was performed following the polypropylene synthesis process using ZN catalysts as presented by Hernández. The process consists of a fluidized bed reactor which is purged before starting the process with nitrogen, followed by feeding 1.2 metric tons per hour of propylene (1.2 TM/h) and 30 g per hour of hydrogen (30 g/h) from the bottom, in addition to 5 kg per hour of catalyst (5 kg/h), 0.24 kg per hour of co-catalyst (0.25 kg/h), one mole per hour of selectivity control agent (1 mol/h), and nitrogen, as shown in Figure 1. This process is discontinuous (batch) and occurs at a temperature of 70 °C and 27 bar pressure. The H_2_S addition is performed in the propylene supply line. The hydrocarbon residues are removed from the resin by means of a stream of nitrogen and steam to obtain a virgin resin [2,36].

For compound analysis, samples were taken at various points during the process (Figure 1). Samples of propylene (1A), nitrogen (2B), hydrogen (3C), and H_2_S (4D) were taken from the feed. In the reactor, samples were taken of the gases that were formed during the reaction (5E), of the catalyst before (7F) and inside (6F) the reactor, and of the unreacted propylene (8H) that was recycled into the process; in the degassing stage, samples were from the PP resin (9I) obtained before removing the hydrocarbon remains, from the gases entrained (10J) by nitrogen and water vapor, and finally from the final PP (11K), as shown in Table 1. For the identification of each substance, the indicative of the sampling point is placed followed by the concentration of H_2_S used (e.g., for propylene in the feed when the concentration of H_2_S is 0.07 ppm, 1A-0.07).

### 2.3. Analysis

The thermal stability and melt index of the obtained samples were evaluated to assess the effects on the final properties. The PerkinElmer TGA7 equipment was used to analyze how the H_2_S content affects the degradation of the PP resin obtained. The TG and DTG curves were obtained by heating 10 mg of the samples at a rate of 20 °C min^−1^ in a nitrogen atmosphere [37,38], with a Tinius Olsen MP1200 plastometer used to measure the melt flow index (MFI) of the PP resins obtained. The samples were heated to 230 °C and the melt was displaced with a 2.16 kg piston, following the norm ASTM D1238-10 [39]. The MFI data were used to calculate the average molecular weight of the samples using the Bremner approximation [40,41].

To verify the concentrations and to know the compounds obtained throughout the synthesis, X-ray fluorescence, Fourier transform infrared analysis, and gas chromatography were used. With the Malvern Axios FAST equipment, the concentrations of sulfur and H_2_S were determined at the different sampling points and X-rays were obtained from the samples placed in the sample holders. A Nicolet 6700 infrared spectrometer (Thermo Scientific) was used with the attenuated total reflectance (ATR) method to analyze the samples, in the form of films of 300 mm diameter and 100 m thickness created by compression molding the PP in a CARVER 3895 hot press [42], to determine the structural changes in the PP matrix caused by the reaction of the catalyst with H_2_S. To ensure the concentrations of H_2_S in the propylene line, an Agilent Technologies 7890B GC-MS was used, and the PP resin samples were studied using the Agilent 7694E headspace sampler with a cycle time of 60 min and an oven at 150 °C [37], following the method of Hernandez [37].

## 3. Results

### 3.1. Polymerization Stage

Polymerization is divided into three stages. The first stage consists of the activation of the propylene double bond and the conformation of an active site through the alkylation of a Ti ion found in the surface layer of –TiCl_3_ [43,44]. In this stage, an intermediate is created by the union of the unsubstituted carbon with the transition metal. This is followed by the monomer polymerization stage, where a coordinate complex is formed between the added monomer, the growing chain, and the catalyst [3]. The last stage is the completion of the polymer chain. For this process, the active complex is deactivated with H_2_ or the elimination of a hydride [44,45]. Since H_2_S can bind to the transition metal present in the active site of the catalyst, its presence in the monomer can inhibit polymerization, with effects on the performance of the process and the final properties of the resins obtained. To study this, the compounds that are formed in each stage are identified and quantified to propose reaction mechanisms.

#### Identification of H_2_S in the Polymerization Stage and Proposed Reaction Mechanism Reaction of H_2_S with TiCl_4_/MgCl_2_

**Figure 2:**
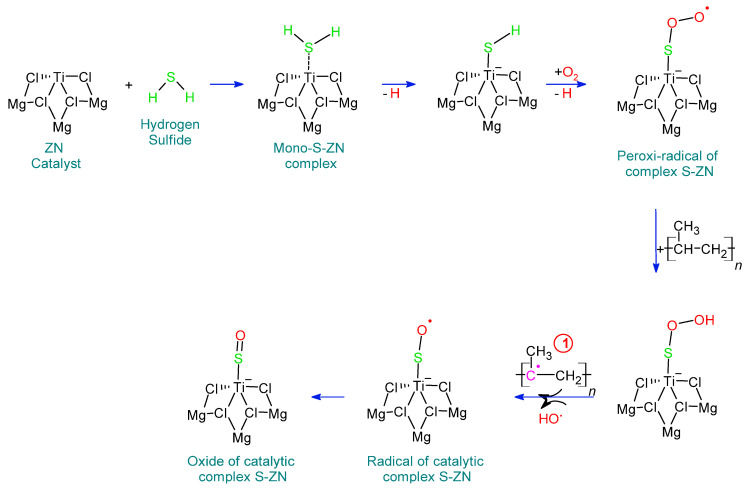
Reaction mechanism of residual H_2_S with TiCl_4_/MgCl_2_.

The H_2_S content varies depending on the stage of the process in which the measurement is made. The first record of H_2_S in the process is at point 4D. This measurement refers to the concentration entered. In a normal process, the presence of compounds at this point indicates that the propylene to be used contains impurities. The next point to look for H_2_S is at the recycle line (8H), since the partial or no presence of a compound at this point indicates that H_2_S is partially or fully reacting with the compounds in the polymerization reactor. The last point where H_2_S is identified and quantified in the polymerization stage is in the reactor (5E). The analysis of this point is supported by the previous measurement and indicates that H_2_S is part of the polymerization reaction. Using the information obtained from these points by FTIR, a reaction mechanism is proposed and is represented in Figure 2.

In the chain propagation stage during PP synthesis, propylene is transferred to Ti-PP, where the olefin is inserted into the PP-alkyl chain [46,47]. This propagation is affected by the presence of impurities that act as inhibitors of different polarities that react with the active Ti center, which depends on the energetic factors: in the case of H_2_S, the coordination with the Ti center on the Ti surface. For MgCl_2_, Ti-H_2_S is favored by 15.1 kcal mol^−1^ [11]. This represents how H_2_S competes with propylene to bind to the active site Ti of –TiCl_3_, interfering with the formation of propylene complexes and their insertion. Where π-complex is initially formed, it coordinates the H_2_S with the Ti of the TiCl_4_/MgCl_2_ complex. This is due to the predominance of the free electrons of hydrogen sulfide over those of propylene that interact with the electropositive Ti [48,49]. The H_2_S-Ti reaction prevails over the formation of -complexes in Ti-propylene since the latter has no barriers and the energy gain is lower. To reverse these interactions, it is necessary to remove the impurities present in the system and resume polymerization [49].

### 3.2. The Degassing Stage

In this stage, the gases present in the resin leaving the reactor are eliminated by purging with nitrogen and steam. At the points that are studied at this stage, it allows us to confirm if H_2_S is adsorbed or absorbed by the PP produced.

#### Detection and Quantification of H_2_S in PP Resin Degassing

**Figure 3:**
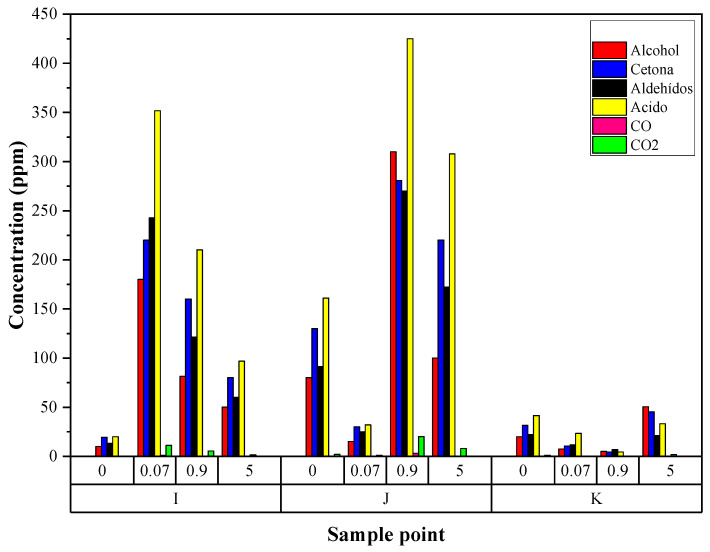
Gases removed in the purge process.

In the degassing of the PP resins, points 9I, 10J, and 11K were evaluated to determine the interaction and presence of H_2_S with the polymeric matrix. Together with the formation of compounds, the compounds and their concentrations are shown in Figure 3. At point 9I, it is determined if the H_2_S remains as a residue in the reactor or if it is transported by the resin when adsorbed or absorbed. At point 10J, samples of the purged gases are obtained, and it is determined whether H_2_S was adsorbed by the matrix, which is the case if the values for H_2_S at this point are nonzero. Finally, point 11K is evaluated. The presence of H_2_S in the polymer matrix indicates that the products obtained are contaminated and have a high probability of not meeting the desired characteristics and specifications, presenting thermal instability, affecting the degradation of the material and the fluid index, among others, so they would become waste. Figure 3 shows how, as the concentration of H_2_S in the system increases, the concentration of gases also increases at the different points sampled as secondary products of the H_2_S-Ti interaction, obtaining compounds such as alcohol in concentrations of 50.2, 81.5, and 180.1, respectively, for the concentrations of H_2_S (0.07, 0.9, and 5 ppm); ketone in concentrations of 80.2, 160.1, and 220.1, respectively, for the concentrations of H_2_S (0.07, 0.9, and 5 ppm); aldehydes in concentrations of 60.2, 121.5, and 242.7, respectively, for the concentrations of H_2_S (0.07, 0.9, and 5 ppm); acids in concentrations of 97.1, 210.1, and 351.7, respectively, for the concentrations of H_2_S (0.07, 0.9, and 5 ppm); and CO_2_ in concentrations of 1.5, 5.5, and 11.3, respectively, for the concentrations of H_2_S (0.07, 0.9, and 5 ppm), for point I, increasing the concentration of these compounds at point J and decreasing at point K. In addition, there was evidence of the presence of 0.2 ppm carbon monoxide (CO) when the concentration of H_2_S is equal to 0.9 ppm and 1.1 ppm of CO when the concentration of H_2_S is 5 ppm at point I.

Compounds such as alcohols, ketones, etc., are products of the Alkoxy-PP-virgin radical that has formed and undergone homolytic cleavage of the single bond adjacent to the carbon of the oxygenated radical. This homolysis forms the methyl-PP-virgin ketone and the radical PP. The radical formed by the catalyst and the H_2_S attacks the tertiary carbon of the methyl-PP-virgin ketone to abstract the hydrogen atom and thus propagate the oxidation of the more stable carbon [50]. This new radical reacts with the oxygen atom to form ketones. On the other hand, the PP radical at the end of the chain reacts with oxygen, giving rise to methanol-PP-virgin which, by abstraction of the tertiary carbon proton of the radical formed by the catalyst and H_2_S, forms an intermediate radical that, by reacting with oxygen, decomposes to form methanol and formaldehyde. Methanol-PP-virgin gives rise to an aldehyde-PP-virgin which, by homolysis of the H–C bond of the terminal carbonyl group, forms the carbonyl-PP-virgin radical, which in turn, by reactions with hydroxyl radicals and hydrogen, gives rise to CO, CO_2_, and formic acid [42].

### 3.3. Influence of H_2_S on the Properties of PP

#### 3.3.1. Effects on the Melt Flow Index (MFI) of PP

**Figure 4:**
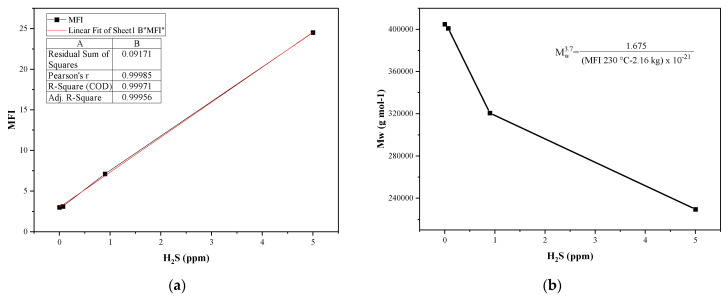
(**a**) The sample melt flow index (MFI); (**b**) the sample molecular weight (Mw).

The melt index allows determination of the molecular changes of the polymer by its relationship with the average molecular weight, which can be affected during polymerization either by incomplete polymerization, chain shortening, or the presence of impurities in the polymer matrix [51,52,53]. Figure 4a shows the values obtained in the fluidity index for each sample, which increases directly proportional to the concentration of H_2_S present in the sample, in a linear correlation with an R^2^ value of 0.9971. This indicates that H_2_S affects the properties of the material by being part of the polymerization reaction through the formation of stable complexes that will form part of the polymer chain. This can be concluded because when studying the catalyst residues (Ti, aluminum, chlorine, and iron), they do not present significant variations, so the effects on the MFI are due to H_2_S [50]. In addition to the variation in the MFI, there is evidence of a change in the molecular weight of the samples, which was calculated using the Bremner formula, yielding the data shown in Figure 4b. The change in molecular weight (Mw) is due to the formation of small chain cuts and the structure’s fracturing. Due to the above, it is expected that the Mw decreases inversely proportional to the MFI [54].

#### 3.3.2. Thermal Degradation of the Material

**Figure 5:**
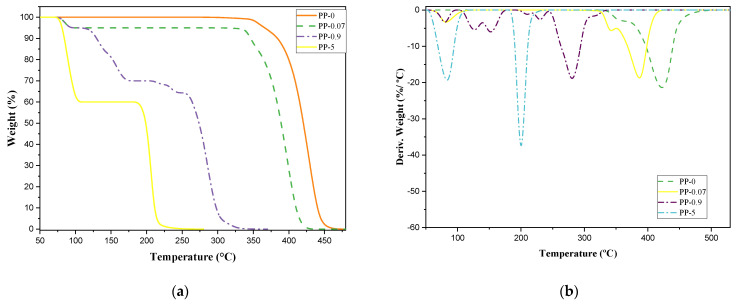
(**a**) TGA of the samples; (**b**) DTG of the samples.

The degradation of the samples was analyzed by means of TGA, where the curves represented in Figure 5 were obtained. It is observed that the thermal stability of the material decreases as the concentration of H_2_S present in the system increases. This change occurs due to the presence of inhibitors such as H_2_S in the polymerization of the material, generating compounds with different functional groups in higher concentrations that evidence the incomplete polymerization of the material and altering the behavior and macromolecular structure of the resins obtained.

When evaluating the thermal degradation of the resins obtained, it is evident that PP-0 and PP-0.07 present similar behaviors, where there is a single drop in weight, which although they are not the same due to the effects of H_2_S, their behavior is constant. Unlike the above, samples PP-0.9 and PP-5 show fluctuations in weight loss, which occurs in two stages. If we compare the weight loss, this also varies very markedly for each sample. Five percent of the weight of each sample occurs at approximately 366 °C for PP-0, at 300 °C for PP-0.07, at 90 °C for PP-0.9, and at 80 °C for PP-5. Reaching 50% of its weight occurs at approximately 420, 390, 275, and 205 °C for PP-0, PP-0.07, PP-0.9, and PP-5, respectively.

## 4. Discussion

The presence of H_2_S in LPG has been studied to predetermine and evaluate the percentage of removal of the sulfuric compounds present in it. However, the effects of this compound on the properties and characteristics of polypropylene have not been studied. To understand how H_2_S affects polypropylene’s properties, it is necessary to study polymerization reactions and how this compound interacts with catalysts and co-catalysts during polypropylene synthesis. The proposed reaction mechanism was performed following two parameters: 1. the interaction between the organometallic compounds and the sulfuric compounds to determine the reaction mechanisms and propose the most appropriate one; 2. using FTIR and chromatography to identify which compounds are produced. These techniques are widely used and are known for their ability to present links and truthful compounds that guarantee the proposed method [55,56,57].

The FTIR study of the resins shows how as the concentration of H_2_S in the system increases, the concentration of gases also increases at the different points sampled, as secondary products of the H_2_S-Ti interaction, increasing its production between 2.09 and 6.03 times the concentration of the gases at the lowest concentration of H_2_S (0.07 ppm) and between 4.6 and 29.45 times when the concentration of H_2_S is 5 ppm. In addition, there is evidence of the presence of carbon monoxide (CO) when the concentration of H_2_S is equal to or greater than 0.9 ppm. This has been found in other studies where the presence of inhibitory compounds promotes the increase of by-products due to interference in the polymerization reaction [58,59,60].

Regarding the changes in the MFI and in the thermal degradation of the material, previous studies have shown that the presence of impurities or non-polymerization compounds affects these two characteristics. The first is due to factors such as chain shortening and polymerization inhibition, which affect the average molecular weight and therefore its melt flow [61,62,63]. These factors in turn influence the decrease in the thermal degradation of the material, which is drastically reduced when the concentration of a compound such as H_2_S increases [38,64,65,66].

## 5. Conclusions

In this study, it is possible to observe the effects of H_2_S on the thermal stability and MFI of PP from the analysis of the reactions of this compound with the active titanium center of the ZN catalyst, followed by oxidation, producing radical complexes of aldehydes, ketones, alcohols, carboxylic acids, CO, and CO_2_ formed by the reactions of the radicals O-O-S-TiCl_4_-MgCl_2_ and (.O-O-S-O-O-)_2_-TiCl_4_-MgCl_2_, decreasing the thermal stability of the material and affecting the MFI that is related to the molecular weight average. The results obtained show that even small traces of impurities in the raw materials can act as polymerization inhibitors when reacting with the ZN catalyst, decreasing its efficiency in addition to affecting the properties of the polymers produced. This can be evidenced by the increase in the production of secondary compounds such as carboxylic acids, ketones, alcohols, and aldehydes. As well as H_2_S, traces of various impurities can be found that can affect the polymer in the same way, reducing the polymerization yield and producing materials that must go through revaluation processes such as pyrolysis. However, it is unknown how the presence of these impurities in the polymer matrix can affect these processes.

## Figures and Tables

**Table 1:** Identification of samples and sampling points.

	Feeding				Reaction				Degassing		
Point	1A	2B	3C	4D	5E	7F	6F	8H	9I	10J	11K
**State of origin**	LPG	Gas	Gas	LPG	Inside the reactor		Catalytic system	Recovered	Exits of reactor	Retired in the purge	Dust
**Substance**	Propylene	N_2_	H_2_	H_2_S	Gases	ZN	ZN	Propylene	PP resin	Gases	PP resin
**ID-0**	1A-0	2B-0	3C-0	4D-0	5E-0	7F-0	6F-0	8H-0	9I-0	10J-0	11K-0
**ID-0.07**	1A-0.07	2B-0.07	3C-0.07	4D-0.07	5E-0.07	7F-0.07	6F-0.07	8H-0.07	9I-0.07	10J-0.07	11K-0.07
**ID-0.9**	1A-0.9	2B-0.9	3C-0.9	4D-0.9	5E-0.9	7F-0.9	6F-0.9	8H-0.9	9I-0.9	10J-0.9	11K-0.9
**ID-5**	1A-5	2B-5	3C-5	4D-5	5E-5	7F-5	6F-5	8H-5	9I-5	10J-5	11K-5

## Data Availability

Not applicable.
